# The effect of extended release tolterodine used for overactive bladder treatment on female sexual function

**DOI:** 10.1590/S1677-5538.IBJU.2016.0303

**Published:** 2017

**Authors:** Athanasios Zachariou, Maria Filiponi

**Affiliations:** 1 Department of Urology, Elpis Hospital, Volos, Greece;; 2 Laboratory of Endocrinology and Metabolic Disorders, Department of Medicine, School of Health Sciences, University of Thessaly, Larissa, Greece

**Keywords:** Urinary Bladder, Overactive, Tolterodine Tartrate, Female

## Abstract

**Introduction:**

Overactive bladder (OAB) is a common condition, especially in middle aged women, requiring long term therapy with anticholinergics to maintain symptoms relief. The aim of the study was to determine the effect of tolterodine extended release (ER) used for OAB treatment on the sexual function of women.

**Materials and Methods:**

Between August 2010 and August 2014, 220 women with confirmed OAB, attended Urogynecology Outpatient Clinic and were prospectively enrolled in this study. 158 women were evaluated, with a comprehensive history, physical examination, urodynamic studies and Female Sexual Function Index (FSFI) questionnaire. 73 patients of group A (control group) received no treatment and 85 patients of group B received an anticholinergic regimen – tolterodine ER 4mg once daily. Data were evaluated again in accordance with FSFI after three months, using SPSS software.

**Results:**

A statistically significant increase was noted in group B in domains of desire (pre-treatment 2.5±0.2 to 4.5±0.2 post-treatment), arousal (3.1±0.2 to 3.1±0.2 respectively), lubrication (3.4±0.3 to 4.3±0.3 respectively), orgasm (3.5±0.3 to 4.5±0.3 respectively), satisfaction (2.6±0.2 to 4.2±0.3 respectively) and pain (2.4±0.2 to 4.6±0.4 respectively) after three months treatment with tolterodine ER. In group A there were no statistically significant changes in pre and post treatment values (p>0.05). Total FSFI score for group B was significantly higher after tolterodine treatment (26.5±1.5) compared to pre-treatment values (17.4±1.4, p<0.01) and to control group A (17.7±1.2 and 17.9±1.5, p>0,05) respectively.

**Conclusions:**

This preliminary study demonstrates that treatment of OAB with tolterodine ER was found to have positive effect on sexual function of patients with OAB*.*

## INTRODUCTION

Overactive bladder (OAB) is a symptom-driven condition characterized by urinary urgency with or without urge incontinence and is usually associated with increased daytime frequency and nocturia (1). It is a common condition, whose prevalence increases with advancing age and compromises health-related quality of life (2). Data from a large study of over 19.000 individuals in four countries across Europe, as well as Canada (the EPIC study), determined that OAB was present in 10.8% of men and 12.2% of women in the general population, becoming increasingly prevalent in individuals aged >40 years, at 13.1% and 14.6% men and women, respectively (3).

The most commonly employed methods for treating newly-diagnosed OAB are bladder training, anticholinergic therapy, beta-3 adrenergic agonists or a combination of them. Modalities such as botulinum toxin injections, sacral neuromodulation and posterior tibial nerve stimulation are showing encouraging results in more refractory patients (4, 5).

Urinary incontinence is believed to contribute to the development of female sexual dysfunction (6). The impact of OAB symptoms on sexual function in women has been evaluated in a few studies (7-12). Kim et al.(8) conducted an Internet survey and found that women with OAB experienced a greater deterioration in sexual quality of life than women with urinary incontinence only.

Female sexual dysfunction is traditionally classified into disorders of desire, arousal, lubrication, orgasm and pain. While epidemiologic data are limited, the available estimates are that 43% of women complain of at least one sexual problem (13). Physiologic, iatrogenic and psychological factors place women at risk for developing female sexual dysfunction (FSD) while lower urinary tract symptoms are an independent risk factor for sexual dysfunction (13, 14).

Tolterodine is a potent, competitive and bladder selective muscarinic receptor antagonist, specifically developed for the treatment of OAB (3,15-17). Clinical trials have shown that tolterodine IR or ER is as effective as oxybutynin in the treatment of OAB, and that it is associated with a significantly lower frequency and severity of adverse events, most notably dry mouth (18). Tolterodine has also been shown to have a beneficial impact on health related quality of life in OAB patients (7).

The aim of the study is to describe the effect of an anticholinergic agent, tolterodine extended release, used for OAB treatment on sexual function of women using a validated questionnaire.

## MATERIALS AND METHODS

Between August 2010 and August 2014, 220 sexual active women, with confirmed OAB, attended Urogynecology outpatient clinic and were prospectively enrolled in this study (19). OAB was defined using the International Continence Society (ICS) definition (19). That is, having urinary symptoms of urgency with or without urge incontinence, with frequency and nocturia.

All women reported having a urination frequency of 8 or more times per day, the presence of urge symptoms that may or may not accompany incontinence, symptom duration of 3 months or longer and no prior history of treatment for OAB. Subjects were excluded from participation if they had active urinary tract infection based on results from urine culture, clinically significant stress urinary incontinence, urinary retention or uncontrolled narrow-angle glaucoma or were at risk for these conditions. Women were also excluded if they had demonstrated hypersensitivity to tolterodine or other component of this product. The research project’s protocol has been approved by the institutional Ethics Committee.

The inclusion criteria also integrated women over the age of 18 years in a sexually active relationship. All participants provided their written informed consent for being included in the study. Women who stated that they were not sexually active were excluded from further analysis. Furthermore, women who were afraid to receive tolterodine in terms of allergy or so, or considered having OAB as a result of aging, were included in control group.Patients were assessed by a comprehensive history, a detailed general and neurological complete physical examination and urodynamic evaluation. Urodynamic techniques and measurements, terms and diagnostic criteria conform to the recommendations of the ICS(19). Symptomatic diagnosis of OAB does not correlate with urodynamic diagnosis of detrusor overactivity (DO) and urodynamic studies are not necessary for the assessment of patients with OAB. The rational of performing themis the differential diagnosis from bladder outlet obstruction, occult neurogenic bladder or other underlying pathology that would weaken the strength of research.

All women were examined employing the pelvic organ prolapsed staging system recommended by the ICS to quantify loss of pelvic organ support (20). Women with more than stage I pelvic organ prolapsed, women who had previously undergone incontinence or prolapse surgery or with neurological diseases were excluded. Women with a history of pelvic muscles training program were excluded because it is accepted that pelvic floor muscles exercises improve female sexual function (21).

The assessment of sexual function and sexual quality of life lends itself to evaluation by patient diary and patient-reported questionnaires (22). According to the latest report of the International Consultation on Sexual Medicine, the Female Sexual Function Index (FSFI) remains the gold standard assessment tool, has a level of evidence 1 and recommendation grade A, for evaluating female sexual dysfunction (23). Item selection and categories were based on the American Foundation for Urological Diseases classification system of female sexual dysfunction. All women were asked to complete the FSFI questionnaire which was previously linguistically validated its Greek version (unpublished data). The FSFI categorizes sexual dysfunction in the domains of (a) desire (b) arousal (c) lubrication (d) orgasm (e) satisfaction and (f) pain. A scoring system is developed to obtain individual domain scores, where higher scores indicate a more healthful condition. Wiegel et al. (24) found that a total FSFI score of 26.5 is the optimal cut score for differentiating women with and without sexual dysfunction.

To determine the eligible women all women were asked to answer the question: “Do you have sexual distress associated with sexual dysfunction?” and only women who gave a negative answer were finally available for analysis, since sexual distress needs special questionnaires to be evaluated. Patients were divided in two groups in terms of their decision to receive tolterodine treatment. In group A, which is defined as control group, 90 women with OAB did not wish to receive any therapy, while in group B, 110 patients with OAB were treated with tolterodine 4mg ER for 3 months.

Patients of group B completed the 3-day micturition diary prior and after the 3rd month of anticholinergic treatment. Patients of group A followed the same pattern prior and after the 3rd month, without anticholinergic therapy. For each episode of urinary symptoms, the patient recorded the date and time of each episode, whether or not they voided, the presence of urgency and/or incontinence, the volume voided, whether or not the episode disturbed the patient’s sleep. 110 patients of group B received tolterodine ER tablets 4mg, once daily, for 3 months. In group A all women attended monthly office visits to ensure that they don’t follow any pharmacotherapy or behavioural therapy for OAB. They were all informed about treatment modalities in their visits.

Voiding frequency, nocturia, urgency episodes, incontinent episodes, number of incontinence pads used and voided volume were measured after treatment using a 3-day micturition diary. Patients from both groups completed the FSFI questionnaire at the beginning and after the completion of the three month’s period, to evaluate their pre and post-treatment sexual function in the case.

Our data were evaluated with the use of SPSS software, USA, release 13.0. The statistical analysis was done using the percentage, paired t-test. Statistical significance was accepted P<0.05. Data are presented as the mean ± standard deviation (range).

## RESULTS

The study characteristics, including age, weight, symptom severity and duration, parity, presence and degree of urge incontinence are presented in Table-1. The incidence of DO was 62.7% in female OAB patients in group A and 64.1% in group B. There was an additional 19.2% in group A and 18.5% in group B that patients revealed DO after provocative maneuvers, such as posture change or coughing. 61% of women with urgency (OAB dry) had DO. Of the 220 women who reported sexual activity the last four weeks, 200 patients agreed to participate and completed the necessary FSFI questionnaires in order to evaluate their sexual function. There were 7 women in group A and 13 women in group B that denied completing the final FSFI questionnaire. Age, body weight, symptom duration, parity, educational level and occupation status were not significantly different between two groups (Table-1).

**Table 1:** Demographic characteristics between two groups.

	Control Group A	Tolterodine Group B	P values
Age (year)	*4*1±6.4years (range 18-48years)	43±8.4years (range 18-51years)	P>0.05
Body weight (kg)	58.5±8.9kg (range 49-78kg)	55.5±7.8kg (range 48-78kg)	P>0.05
Symptom duration (year)	4.3±3.1years (range 0.3-6years)	3.9±3.1years (range 0.3-6years)	P>0.05
Parity	2.1±1.2 (range 0-4)	2.0±1.3 (range 0-4)	P>0.05
**Level of education**	Educated: 70	Educated: 83	P>0.05
	Not educated: 3	Not educated: 2	
**Occupation status**	Occupied: 50	Occupied: 60	P>0.05
	Not occupied: 23	Not occupied: 25	

None of them stated being in menopause. In group B, throughout the study, 12 patients discontinued tolterodine medication for the following reasons: three for dry mouth, two for insufficient therapeutic response, three because of low treatment compliance and four because they realized that OAB requires a long term therapy. In Group A (control group), ten patients discontinued because during the first month office visit decided to follow a behavioral therapy and receive an anticholinergic regimen.

Mean total FSFI and its subscales were significantly different after three months between two groups. Repeated statistical analysis showed that mean FSFI and its subscales were significantly different before and after treatment in group B (P<0.01), but not in control group A (Table-2).

**Table 2:** Mean FSFI (total and subscales) pre and post-treatment in Group B.

	Pre-treatment	Post-treatment	p values
Desire	2.5±0.2	4.5±0.2	P<0.01
Arousal	3.1±0.2	4.4±0.3	P<0.01
Lubrication	3.4±0.3	4.3±0.3	P<0.01
Orgasm	3.5±0.3	4.5±0.3	P<0.05
Satisfaction	2.6±0.2	4.2±0.3	P<0.01
Pain	2.4±0.2	4.6±0.4	P<0.01

**Total FSFI score**	**17.4±1.2**	**26.5±1.5**	**P<0.01**

Thirty five women (41.7%) complained mainly for sexual pain disorders, 23 (27.4%) for hypoactive sexual desire, 13 (15.5%) for sexual arousal disorders and 13 (15.5%) for lubrication disorders and orgasmic deficiency.

After three months tolterodine treatment mean frequency, nocturia and incontinence episodes decreased statistically significant (Table-3) in comparison to control group, which showed no statistically significant differences.

**Table 3:** Evaluation of urinary parameters in the tolterodine-treated group.

	Pre-treatment	Post-treatment	P values
Frequency	11.93±2.58	8.99±1.54	P=0.041
Urgency episodes	6.78±3.58	4.38±2.14	P=0.032
Nocturia	1.43±1.04	0.6±0.4	P=0.045
Incontinence episodes	2.21±0.95	1.2±0.1	P=0.009
Incontinence pads	4.91±0.95	1.89±0.57	P<0.001
Voided volume (mL)	110±35	145±42	P=0.009

Furthermore 53% of tolterodine patients who were incontinent at baseline became continent by the study endpoint. In accordance with the improvement in symptoms, other objective measurements such as the mean volume voided per micturition increased significantly during the study (35mL) compared to the women before treatment (p<0.010). The number of incontinence pads used by patients significantly reduced after treatment (p<0.001).

The total FSFI score was significant higher (26.5±1.5) compared to pre-treatment values (17.4±1.4) (p<0.001) although there was a residual sexual dysfunction mainly in subscales of lubrication and orgasm (Table-2).On the other hand, in control group A there were no statistically significant changes after three month’s observation. More specifically,pre and post-treatment values, expressed with median value ± standard deviation, were for desire 2.5±0.3 and 2.6±0.2 respectively,for arousal 3±0.3 and 3±0.4 respectively, for lubrication 3.6±0.3 and 3.4±0.3 respectively, for orgasm 3.4±0.1 and 3.5±0.2 respectively, for satisfaction 2.8±0.3 and 2.7±0.2 respectively and for pain 2.5±0.3 and 2.4±0.3 respectively. Moreover, the pre and post-treatment total FSFI score for group A was 17.7±1.2 and 17.9±1.5 respectively. All P values were >0.05 implying that there were no statistically significant changes during the three month’s observation.

## DISCUSSION

A significant amount of new information has been made available regarding the effects of OAB on female sexual function and quality of life (7, 8). Treatment of cases with OAB includes behavioral, pharmacological, surgical interventions and peripheral electrical stimulation. It seems logical that medical treatment of OAB improves sexual function of women with OAB. However, the amount of data, related on the effect of antimuscarinic agents, used for OAB treatment on sexual function of women is insufficient in the literature. This study aimed to describe the effect of tolterodine 4mg ER, specifically used for OAB treatment on sexual function of women.

Female sexual health is a dynamic and multifaceted phenomenon that is closely linked to a woman’s overall quality of life. Sexual dysfunctions can interfere with intimacy, affect a marital relationship, and ultimately erode well-being and overall health. In contrast to the burgeoning data on men, clinical trials on sexual dysfunctions in women are few and also sexual dysfunctions are likely more common in women than in men.

Temml et al. (26) reported that 25% of incontinent women had some form of sexual dysfunction, and most of their subjects believed that incontinence during coitus was the most bothersome symptom. Other investigators (10, 27, 28) have found that detrusor overactivity has more impact on sexual function than urodynamic stress incontinence while others found that 1 to 4 women with OAB report some form of sexual impairment (20 [Bibr B25], 28). Yip et al. (10) found that women with urodynamic stress incontinence and detrusor overactivity had poorer quality of life and sexual satisfaction. Regression analysis revealed that poor sexual satisfaction correlated with worsening marital relationships in the incontinent women in their study. More than 60% of incontinent women that reported sexual pain disorders reported also recurrent bacterial cystitis that could be implicated with inflammation at the genitalia, flogosis, vaginal lubrication disturbance and higher incidence of pain disorders (29).

Some researchers believe that lower urinary tract symptoms are more important with respect to sexual activity and sexual satisfaction than urine leakage during intercourse and that women with OAB complained mainly of repeated urgency or frequency during intercourse (8). According to the participants the incontinence associated with intercourse does not have the greatest impact but that urgency and frequency after coitus as well as the fear of leakage during stimulation and intercourse are quite detrimental to enjoying sexual relations.

The results of the current study revealed that tolterodine ER applied for OAB treatment improved female sexual dysfunction. The aspects of sexual life that improved in this study were pain, orgasm, sexual enjoyment, sexual desire, and even vaginal wetness. We believe that this improvement in the sexual quality of life comes from the major improvement described in bladder pain. This finding may be in favor of a causal relationship between urinary symptoms and sexual dysfunctions. These results may not be true for women with OAB who are not sexually active.

In OAB patients, pain could be derived from vaginal dryness and lack of lubrication caused by the urine presence in the vagina that affects the normal acidic pH of the vagina and the normal flora as well as hypertonicity of pelvic floor muscles due to fear of urine leakage(8). Sexual pain disorders may be due to long term effects of recurrent infection and inflammation of the genitalia. Furthermore in the arousal phase there is an increase in clitoral and vaginal blood flow(30). The decreased localized blood flow may facilitate reduced bladder wall resistance to bacteria and a loss of genitalia excitability (29).

In a previous study of Eftekhar et al. (4), fifty women facing OAB, were randomly assigned to PTNS (posterior tibial nerve stimulation) plus tolterodine or tolterodine alone treatment. The results showed no significant difference between two groups regarding FSFI score and its subscales. Hajebrahimi et al. (7) indicated an improved sexual function of women suffering from OAB, by administrating tolterodine IR, using the Arizona Sexual Experience Scale (ASEX).

The impact of OAB or urinary incontinence on sexual health is not a topic that patients freely initiate. Not only may women, who experience incontinence and sexual impairment, be embarrassed to approach a health professional, but health professionals may also be embarrassed to confront patients. The use of self-administered questionnaires provides a means of collecting information on sexual health while reducing potential embarrassment and response bias associated with interviewer-administered questionnaires.

In conclusion, according to our research, treatment of OAB with tolterodine ER improves sexual function of female patients with OAB. However, this study has some limitation. First, we were not able to observe whether adverse effects of the medication, such as dry mouth and constipation, affect sexual function because these patients were withdrawn and did not complete the final FSFI questionnaire. Second, two only patients presented total FSFI score after treatment worse than the introductory FSFI total score. The limited number of these patients did not permit us to conclude which are the factors for not having good response in sexual function.

Our study had a control group with women not taking anticholinergic treatment but no placebo group.Thus, there is no evaluation about the effect of placebo on sexual function. Furthermore, there was no power analysis for confirming the validity of our sample size. It is not possible to use a randomization method in our paper since the decision of female patients, to receive tolterodine or not, was the reason for being in group A or group B. Someone could claim that it is a random process of selection although this can by chance lead to disparities. In spite of these limitations, we believe that significant results have been obtained, which are of value for clinicians working in this field. Women with OAB should be evaluated also in terms of sexual function to provide better quality of life and the therapeutic use of tolterodine ER for OAB improved FSFI total scores of the patients.
